# Effects of Aspirin on Colorectal Cancer Related to Lynch Syndrome

**Published:** 2012-11-01

**Authors:** Sarah Leilani Beck,

**Affiliations:** From Binghamton Gastroenterology Associates, Binghamton, New York

## Abstract

This article is a review of "Long-term effects of aspirin on cancer risk in carriers of hereditary colorectal cancer: An analysis from the CAPP2 randomised controlled trial" by Burn et al. (2011), Lancet, 378, 2081–2087. For another perspective on the Burn et al. article as well as a discussion of challenges faced when interpreting a large prospective trial, please see the related article by Rita Wickham.

Colorectal cancer (CRC) is the second leading cause of cancer-related death in the United States (Centers for Disease Control and Prevention [CDC], 2012) and remains the third most common cancer in men and in women (American Cancer Society [ACS], 2012a). The American Cancer Society estimates that 103,170 new cases of colon cancer and 40,290 cases of rectal cancer are expected to occur in 2012.

With the use of colorectal cancer screening tests such as colonoscopy that allow the detection and removal of colorectal polyps before they progress to cancer, the incidence rates of developing colorectal cancer have been declining over the past 2 decades. According to CDC statistics, the overall incidence of CRC decreased by 3.0% per year among men and 2.4% per year among women between 1999 and 2008 (CDC, 2012). Despite the overall decrease, CRC rates have been increasing by 1.7% per year since 1992 among young adults considered at average risk: those under the age of 50 and for whom screening colonoscopy is not recommended (ACS, 2012b).

## Lynch Syndrome

Hereditary nonpolyposis colorectal cancer (HNPCC), also known as Lynch syndrome, is a type of autosomal dominant, inherited cancer of the colon and rectum that poses significant risk for CRC. Lynch syndrome also carries an increased risk of cancers affecting the stomach, small intestine, gallbladder, hepatobiliary ducts, skin, brain, and urinary system (including the ureters and kidneys). Women with Lynch syndrome have a higher risk of endometrial and ovarian cancer. Individuals with Lynch syndrome can develop colon polyps at an earlier age than the general population, and polyps that do develop are more likely to become cancerous (National Library of Medicine [NLM], 2008). However, polyps are not thought to develop in greater numbers for individuals with Lynch syndrome vs. those in the general population. Approximately 2% to 5% of colorectal cancer cases in the United States are related to Lynch syndrome (Medscape, 2011).

Sequence variation or mutations in any one of the *MLH1, MSH2, MSH6,* and *PMS2* genes increase the risk of developing Lynch syndrome. These genes are all involved in mismatch repair (MMR) that can be made during DNA replication. The MMR genes produce a protein that is responsible for recognizing and correcting pairing mismatches. Cell cycle progression is constantly monitored to ensure that the correct sequence of cell division is achieved and that DNA-damaged cells do not replicate. Apoptotic response (programmed cell death) is dependent on the recognition and processing of DNA damage by the MMR genes (O’Brien & Brown, 2006). If a mutation occurs in any of these genes, abnormal cells can continue to divide unchecked, and accumulated mutations can lead to uncontrolled cell growth and potential cancer (NLM, 2008). Mutations of DNA damage-repair genes play a role in predisposing colorectal epithelial cells to mutations and potentially influencing the rate of tumor growth or type of pathologic change in tumors (National Cancer Institute [NCI], 2012).

## Colorectal Cancer Screening

Current guidelines for detecting early-stage CRC focus on screening, such as the recommendation of colonoscopy beginning at the age of 50 in average risk individuals, prior to the presence of symptoms. Screening colonoscopy allows for the detection and removal of colorectal polyps that have the potential to become cancerous. As stated previously, with the increased incidence of CRC in younger adults, timely evaluation of symptoms regardless of age is important.

In high-risk individuals with Lynch syndrome or based on family history of Lynch syndrome, current American Cancer Society guidelines recommend screening colonoscopy beginning at age 20 to 25, or 10 years prior to the age cancer was diagnosed in the youngest affected family member—whichever is earlier. Colonoscopy should then be done every 1 to 2 years (ACS, 2012b). With the increased risk associated with inherited genetic conditions such as Lynch syndrome, sometimes screening is not enough, and alternative methods of prevention before the onset of cancer become the focus.

While colonoscopy and polyp removal have made strides in reducing the overall incidence of CRC, the use of aspirin in the prevention of CRC has also been investigated. Recently, a study entitled "Long-term effects of aspirin on cancer risk in carriers of hereditary colorectal cancer: An analysis from the CAPP2 randomised controlled trial" (Burn et al., 2011) was published in *The Lancet*. Unlike previous studies that looked at reduced risk of adenomas with the use of aspirin, this most recent study examined patients with HNPCC/Lynch syndrome, who are known to have a high risk of developing CRC.

The Colorectal Adenoma/carcinoma Prevention Programme (CAPP) is a program of genetically targeted randomized controlled clinical trials that have the primary aim of determining whether taking certain supplements on a daily basis would reduce the initiation and progression of adenoma/cancer in a genetically predisposed population (CAPP, 2005). The rationale for aspirin/nonsteroidal anti-inflammatory drug (NSAID) use as a chemoprevention strategy comes from the effort to prevent or delay the development of cancer by taking medicines, vitamins, or other agents. This approach is thought to prevent the formation of adenomas and may prevent cancer (NCI, 2012). The mechanism of action of aspirin/NSAID irreversibly inactivates both COX-1 and COX-2, blocking the synthesis of prostaglandins. Prostaglandins are potent mediators of inflammation. It is thought that NSAIDs may exert their chemopreventive effects in the colon by restoring a normal frequency of apoptosis (NCI, 2012).

## Overview of the CAPP2 Trial

The CAPP2 trial targeted carriers of Lynch syndrome and looked at whether daily ingestion of aspirin and/or resistant starch reduced the initiation and progression of adenoma. Study subjects had to be either proven carriers of pathologic mutations in MMR genes or have family members with Lynch syndrome and have had at least one of the following: colorectal cancer, a related carcinoma, an adenoma of over 5 mm in diameter, an adenoma at younger than 40 years old, or a confirmed adenoma of any size at more than 1 endoscopy. The study subjects were over 25 years old with no upper age limit; they had to have an intact colon or have had only a segmental resection, as well as normal nonmedicated bowel movements with three or fewer formed stools a day. Participants were given 600 mg of aspirin or 30 g of treatment starch (equivalent to 13.2 g of resistant starch), neither, or both (CAPP, 2005). The four arms of the trial were aspirin/treatment starch, placebo tablet/treatment starch, aspirin/placebo starch, and placebo tablet/placebo starch.

The study involved almost 1,000 patients from 43 international sites. Of the total number of participants, 76 were not randomized due to aspirin sensitivity or history of peptic ulcer disease. A total of 427 subjects were assigned to 600 mg of aspirin per day, with the other 434 receiving a placebo. The primary outcome was the development of CRC, and the secondary outcomes were development of colorectal adenomas and/or the development of other Lynch syndrome–related cancers. Outcomes in both groups were followed for a minimum of 2 years; 258 patients on the aspirin arm and 250 on placebo were followed for 2 years or longer.

## Results

A total of 48 patients developed CRC. Of the participants who had taken aspirin for at least 2 years, there was a statistically significant difference (*p* = .05) in cancer incidence reported between the two groups: 30 individuals in the placebo group developed colorectal cancer vs. only 18 in the aspirin group. Of those diagnosed, 40% were Dukes’ stage A, 19% Dukes’ B, 19% Dukes’ C and D, and 8% were unknown. The majority of tumors (51%) were located in the ascending colon, transverse colon, and splenic flexure, and the remaining were located in the descending colon (11%), the sigmoid colon and rectum (23%), and in unknown locations (15%). No significant group differences existed in staging between the aspirin and placebo groups. Evaluation of reduced cancer incidence in the study was at 55.7 months in patients with Lynch syndrome.

The overall hazard ratio (HR) of developing CRC was 0.63 (95% confidence interval = 0.35–1.13, *p* = .12) favoring the aspirin group. For those who took aspirin and completed 2 years of follow-up the HR was 0.41 (0.19–0.86, *p* = .02), indicating that those who took aspirin were 59% less likely to be diagnosed with CRC. Adverse events did not differ between the two groups during the intervention trial.

## Discussion and Implications

The mechanism of action of aspirin as chemoprotection remains unknown, although in theory it is thought to be related to the ability of aspirin to interrupt the carcinogenic pathway (American Gastroenterological Association, 2009). In a 1998 study looking at aspirin and hereditary nonpolyposis, it was shown that microsatellite instability in colorectal cancer cells in HNPCC (specifically looking at cells deficient for a subset of the human mismatch repair-MMR genes of hMLH1, hMSH2, and hMSH6) was thought to be markedly reduced during exposure to aspirin (Rüschoff et al., 1998). Aspirin and other NSAIDs are thought to be cancer preventives related in part to antiproliferation- and apoptosis-inducing activities. In other words, aspirin is thought to promote programmed cell death and in theory inhibit the formation of abnormal cells at increased rates, as can occur in cancer formation.

From a clinical standpoint, the CAPP2 trial demonstrates convincing evidence for the use of aspirin in patients with Lynch syndrome (Burn et al., 2011). While aspirin can be considered in this high-risk population in conjunction with continued intensive cancer surveillance (frequent colonoscopies every 1 to 2 years in the HNPCC/Lynch syndrome population; Chan & Lippman, 2011), the risks of aspirin use must be carefully considered. From a gastroenterology standpoint, the potential for GI complications with the use of long-term high-dose aspirin may still outweigh the chemoprotective benefit in preventing CRC.

Some limitations of the CAPP2 trial are that the optimal duration of aspirin for benefit is still not known, the age at which to start aspirin has not been investigated, the dose of aspirin used in the trial is not available in the US, and toxicity/complication data are not well discussed or reported. Patients were enrolled in the study with clinical Lynch syndrome, but genotype was not officially confirmed. Currently, genetic testing is available to identify mutations in the MMR genes; this testing should be strongly considered in subsequent studies. Further clinical discussion is needed with regard to this risk/benefit issue.

The complications of long-term aspirin use and the potential issues of GI bleeding and need for transfusions, hospitalizations, and overall burden on health outcomes should be considered as well. In a study published by Huang, Strate, Ho, Lee, and Chan in 2011, regular aspirin use was associated with GI bleeding, and risk was thought to be more strongly related to the increased dose of aspirin rather than the duration of aspirin use (Huang et al., 2011).

Although the Burn et al. study shows compelling evidence to consider the use of aspirin to prevent colon cancer in individuals with Lynch syndrome, further studies are needed to clarify the aspirin dosage and duration of therapy. In the United States, aspirin is not available in the 600-mg strength. There is need for further research, and subsequent studies should compare the effect of different aspirin doses in Lynch syndrome.

## Conclusion

The Burn et al. study has come at a time when chemoprevention needs to be strongly considered as an adjunct to current surveillance in the prevention of CRC in high-risk populations such as individuals with HNPCC/Lynch syndrome. Further conclusive studies and clinical discussions are warranted, with the hope that dosage and duration recommendations can be made and applied to patient care.
